# Account of a Specimen of Partial Fracture of the Neck of the Thighbone: And Remarks on the Proper Source of Nutrition of the Head of the Bone

**Published:** 1846-02

**Authors:** T. Wilkinson King


*Account of a specimen of Partial Fracture of the neck of the thigh-bone : and Remarks on the Proper Source of Nutrition of the Head of the Bone.* By T. Wilkinson King, Esq.— Surgeons are aware, that the inferior part or buttress of the cervix femoris, or that part between the head and the trochanter minor, is by far the most solid. All the super-incumbent weight is directed on this part, and thus, it would seem, its comparative hyper-nutrition is excited or induced; it happens, however, that, with general senile atrophy, and loss of elasticity and agility, this part is particularly prone to give way ; the head of the bone sinks into the dense shell, and the inferior fragment is driven up into the cancelli of the head. At the same time, less violence is done to the thin elastic shell of the upper part of the neck of the femur, or that between the shell and the trochanter major. Possibly it is not a very rare event that in this way fracture extends through somewhat more than half of the neck. Considering the fact of partial fracture of the neck as sufficiently established, I wish to connect it with the peculiar mode of nutrition of the head of the femur. The artery which supplies the head of the femur, while it constitutes an epiphysis, is persistent through life. It is a large terminal branch of the internal circumflex artery, which enters a foramen a little below and behind the highest point of the neck of the femur. After this it curves over the denser layer of cancelli, left by the union of the epiphysis to the shaft, directing its course beyond the insertion of the round ligament, to which, I doubt not, it furnishes nourishment.

Now, it is remarkable, that this vessel occupies the situation of the greatest immunity from violence ; and that if only a little periosteum about it escape division when complete fracture occurs, it may be left entire to sustain that which I think could scarcely live without it. This consideration seems corroborated by all the examples I have examined of ligamentous union after fracture at this part. Whether there be a re-union by solid ligament, by a few scattered bands, or by a kind of capsule and cell, (all rare events,) I find the course of this vessel apparently uninterrupted.

It is needless to say, that these observations apply to the doubtful cases of bony re-union of the cervix femoris. I would add, there may be more reasons against bony union at this part than have yet been considered. The fracture is often as much the result of atrophy as of violence, and the atrophy proceeds after the fracture has taken place. The position of the head in a good variety of supposed specimens of united fracture, indicates the course of alteration I have pointed out; the pit of the head, instead of presenting upwards and inwards, faces inwards, or inwards and downwards; evidently showing that the upper connections or relations of the head have been much less changed than the lower or inner. Nature can but feebly, and rarely, and perhaps never, make any efficient effort to re-unite fractures which separate the head of the femur from its basis.—Abridged from *Guy's Hospital Reports*—*Lond. and Ed. Monthly Journ. Med. Science.*

